# Assessing impacts of rainfall intensity and slope gradient on runoff process and dissolved organic carbon loss via surface flow and interflow under simulated rainfall

**DOI:** 10.1371/journal.pone.0328611

**Published:** 2026-01-23

**Authors:** Lu Xu, Jun Lu, Dan Zhang

**Affiliations:** 1 School of Software Engineering, Chengdu University of Information Technology, Chengdu, Sichuan, China; 2 Institute of Mountain Hazard and Environment, Chinese Academy of Sciences, Chengdu, Sichuan, China; Shandong University, CHINA

## Abstract

Dissolved organic carbon (DOC) is an indispensable component of the global carbon cycle. Previous research on runoff process and DOC loss mainly focused on surface flow, with few reports of the hydrological pathway of interflow or DOC loss via interflow. To address this deficiency, a series of rainfall simulations were conducted with three rainfall intensities of 60, 90, and 120 mm h^-1^ (R60, R90, and R120) and three slope gradients of 5, 15, and 25° (S5, S15, and S25) of purple soil. The initial time of surface flow was faster under high rainfall intensity and steep slope, and the initial time of interflow increased with increased rainfall intensity under gentle slope. In general, the surface flow rates increased first, and reached a steady state within 10–35 min. The interflow curves were single-peak curves for R60-S5 and R90-S5, but exhibited a continued rising trend for other treatments. The interflow volume occupied 69.2% of the total runoff volume under R60-S5, and the percentages of interflow decreased as the rainfall intensity and slope increased. These results indicated that interflow was an important hydrological pathway of purple soil. The DOC concentration of the surface flow decreased with rainfall duration, with opposite trend for DOC concentration of interflow. The DOC concentrations in the interflow were 1.35–2.34 times higher than those in the surface flow. However, the rainfall intensity and slope had little effect on DOC concentrations in both surface flow and interflow. Furthermore, the DOC loss fluxes via surface flow and interflow were 3.77–26.94 g and 0.41–13.73 g, respectively, and the ratios of interflow DOC loss fluxes to the total DOC loss fluxes gradually decreased with the increase of rainfall intensity and slope. Under R60, DOC loss via interflow was the major DOC loss pathway, accounting for 51.0%−78.4% of the total DOC loss, whereas for R90 and R120, DOC loss via surface accounted for >90%. Moreover, runoff volume was positively linearly correlated with the corresponding DOC loss fluxes in both the surface flow (R^2^ = 0.93, *P* < 0.01) and interflow (R^2^ = 0.99, *P* < 0.01). These results provide a scientific basis to estimate the fluxes of DOC loss and control carbon loss in the purple soil area of China.

## Introduction

Soil organic carbon (SOC) plays an essential role in maintaining soil quality, land ecosystem services, and the carbon cycling [1–3]. Soil erosion, especially water erosion, can lead to soil loss and also affect dynamic changes of SOC [4,5], resulting in declining land productivity, increasing nonpoint-source pollution, and is part of the global carbon cycle [6,7]. Water erosion in China displaced 180 ± 80 Mt C yr^-1^ of soil organic carbon during the last 20 years, which caused a redistribution of land atmosphere CO_2_ fluxes [8]. Soil carbon losses by water erosion is a potentially critical contribution to the global carbon cycle [9]. Among various SOC fractions, dissolved organic carbon (DOC) is one of the most active and mobile carbon pools and consequently plays an important role in the global carbon cycle [10]. Previous studies have mostly considered the dynamic of particulate carbon (POC) associated with sediment [11–13], but DOC associated with runoff is also an indispensable component of global carbon budgets [1,14,15]. In addition, previous studies of soil DOC dynamics mainly focused on migration driven by surface flow [15–19]. However, for well-developed interflow soils, the hydrological pathway of DOC loss via interflow cannot be ignored in evaluating DOC loss fluxes [10,19–23]. Thus, studies are needed to quantify the DOC loss from both surface flow and interflow.

Previous studies have demonstrated that interflow can represent a substantial portion of the total DOC loss in runoff. For instance, in the sloping lands of the Sichuan Purple Soil region, Li et al [19] reported that interflow was a crucial pathway for lateral DOC transport during the rainy season, with annual DOC fluxes of 565.5, 802.1, and 1090.1 mg·m ⁻ ² over three consecutive years. Hua et al [10] conducted a free-drain lysimeter experiment of Regosols in Southwest China, and found an annual loss flux of DOC through interflow of 865.5 mg·m^-2^, which was 92 and five times higher than DOC loss fluxes through sediment and surface flow, respectively. Many factors affect the characteristics of soil DOC loss, including rainfall intensity, slope gradient, soil type, soil properties and agricultural land management [14,15,21]. Li et al [11] studied four typical loess soils and found that sediment and surface flow are the main carriers of SOC, accounting for SOC losses of up to 90%. However, Wang et al [22] and Fu et al [23] reported that interflow, rather than surface flow, could be the main pathway of nutrient loss for purple soil and weathered granite slopes. Gaelen et al [17] identified rainfall intensity as the main factor controlling DOC loss with runoff because it is the main driving force of runoff. Ma et al [18] found that rainfall intensity had a bigger effect on DOC lateral transport than on DOC vertical mobilization, and the DOC loss increased with increased intensity rainfall in the red soil region of Hunan province in China. Slope gradient also exerts strong effects on DOC loss [24]. Fei et al [25] carried out artificially simulated rainfall experiments for red soils, finding that the total organic carbon loss of surface flow first increased and then decreased with increasing slope gradients. However, there was a continued rising trend as slope gradients increased in interflow. Wang et al [22] found that nitrogen loss in the surface runoff and sediment yield increased as the slope gradients increased, and found the opposite trend regarding nitrogen loss in interflow.

Purple soil, covers a total area of 1.6 × 105 km^2^ in the Sichuan Basin, in southwestern China, and is one of the most important soil types for agricultural production in the upper reaches of the Yangtze River [26,27]. Purple soil is classified as Eutric Regosols (according to FAO Soil Taxonomy) and is developed from the products of the rapid weathering of underlying sandstones, siltstones, and mudstones. This weathered soil is characterized by a thin soil layer, low organic carbon, and high soil erodibility. Due to the relative thin soil layer and typical “soil-bedrock” dual structure, the soil layers can be easily saturated by rainfall [28]. These soils also have high permeability, with extensive interflow through the “soil-bedrock” interface during the rainy season [26,29]. Most studies of purple soil were carried out in the field under natural rainfall conditions, limiting conclusions about the lateral transport of DOC via surface flow and interflow due to the many uncontrollable factors of natural rainfall. To more carefully probe the contributions of surface flow and interflow to DOC loss, we conducted an indoors rainfall simulation experiment with controlled rainfall intensity and slope gradient. The objectives of this study were as follows: (1) to investigate the dynamic changes of runoff rate and DOC concentration; (2) to quantify the distribution of DOC loss via surface flow and interflow.

## Materials and methods

### Soil collection and preparation

**Fig 1:**
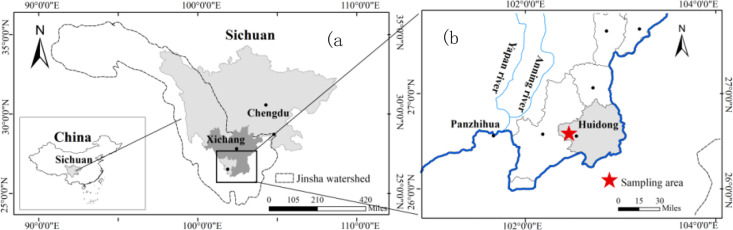
Location of sampling area. (a) Overview map of China and Sichuan province showing the broader regional context. (b) Detailed map of the study area in Sichuan province, with the sampling area marked by a red pentagram. The map was created using QGIS, and includes a north arrow and scale bar.

The soil used in this study was collected from Liangshan Yi Autonomous Prefecture (26°33′42″N, 102°27′53″E), in the southwest of Sichuan Province, China (Fig 1). This region is dominated by a typical subtropical monsoon climate [30], with an average annual temperature of 16.2°C, and mean annual precipitation of 1111.6 mm, with about 95% of precipitation concentrated in the summer rainy season [27]. This region is primarily purple soil, with 35 ~ 45 cm soil tillage layer thickness. A large quantity of soil samples were collected from a single, uniform source area at once to minimize inherent variability. Soil samples were collected every 5 cm from the surface to 40 cm deep, and soil bulk density was determined by the cutting ring method. The soils from different layers were packed separately and transported to the laboratory. To maintain the natural state of the soil, no sieving was used [31]. We mixed the soil material of the same layer thoroughly, removed the coarse stones and organic debris, and then air-dried the tested soil to achieve a water moisture of 10% ± 1%.

### Soil flume and preparation

**Fig 2:**
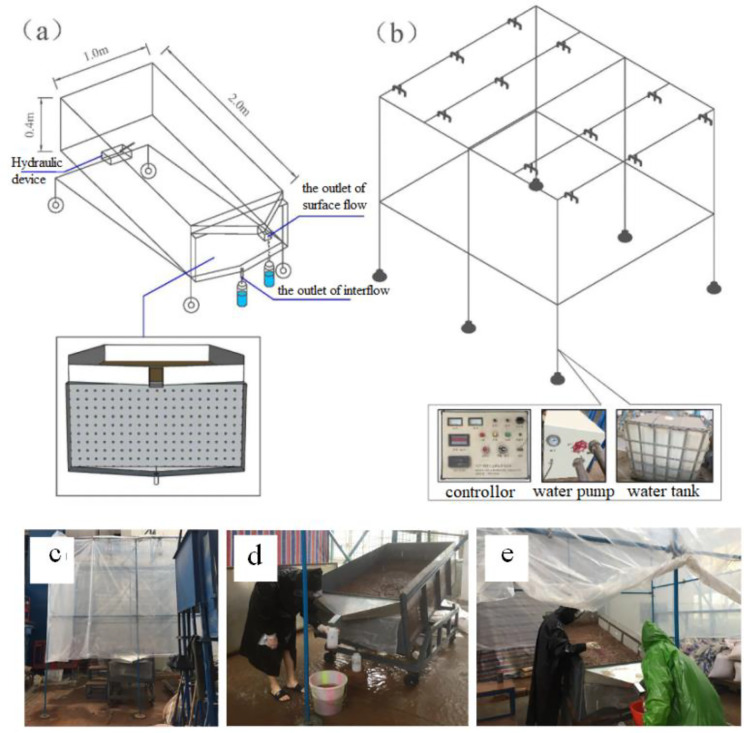
Schematic diagram and overview of the artificial rainfall simulation system. (a) Soil flume containing the experimental soil. (b) The central control equipment for the rainfall simulation experiment, including controllor, water pump and water tank. (c) Rainfall simulator. (d) Runoff collection flumes. The “V-shaped” trough was used to collect the surface flow and sediment, and the outlet at the bottom of the soil flume was used to collect interflow. (e) A photograph of the entire experimental process in operation.

A three-dimensional soil flume (2.00 m × 1.00 m × 0.40 m, length × width × height) was constructed of steel sheets (Fig 2). The slope gradient of the soil flume can be adjusted from 0–30° by hydraulic device. The bottom of the soil flume was sealed to simulate a natural impermeable layer of a “soil-bedrock” interface. A “V-shaped” trough installed at the downslope edge of the flume was used to collect the surface flow and sediment, and the outlet at the bottom of the soil flume was used to collect interflow. Before filling the soil flume, a 5-cm layer of thick gravel and then gauze were put on the bottom of the flume. Next, a 35-cm soil layer was put on top of the gravel layer. To ensure the uniformity of the soil layer, the soil applied in 5-cm increments, with a soil bulk density of 1.3 g cm^-3^. In total, seven successive 5-cm soil layers were added to the soil flume. During filling, the soil was gently compacted in each layer to diminish aggregate breakdown and to obtain the desired bulk density, and the edges of the soil flume have also been compacted [32,33]. To diminish the discontinuity between layers, the surface of each layer was smoothed and then roughened to allow the adjacent two layers to tightly combine [13]. Additionally, new soil was used for each rainfall simulation event, rather than keeping the same soil in the flume for a series of rainfall simulation events.

### Rainfall simulator

To make the raindrop size and the distribution of the simulated rainfall closely resemble that of natural rainfall, a down sprinkler artificial rainfall simulator system (NYJL-10, Nanjing Forestry University) was employed in this study. The rainfall simulator included twelve sets of rainfall shower heads (three nozzles per head), and rainfall was applied at a rainfall height of 6 m. A schematic diagram of the soil flume and rainfall simulator is shown in Fig 2. By changing both nozzle size and water pressure, the available rainfall intensity could be adjusted from 10 to 240 mm h^-1^ with uniformity of 88%. While the heterogeneity may contribute to variability in runoff generation and DOC mobilization, the randomization of treatments and the integration of measurements over the entire plot area ensure that the reported results reflect representative average responses to the applied treatments. The rainwater applied in the experiments was prepared using laboratory tap water (treated municipal drinking water). The DOC content of this water source was consistently low and considered negligible. The same water source was used for all replications to ensure consistent initial DOC conditions. Prior to the rainfall simulation experiment, the rainfall intensities were calibrated using a gauge and four rainfall barrels distributed around the soil flume.

### Laboratory rainfall simulation experiments

The rainfall simulation experiments were carried out using the soil flume and rainfall simulator in the Key Laboratory of Mountain Hazards and Surface Process, Institute of Mountain Hazards and Environment, Chinese Academy of Sciences, Chengdu City, China. According to the classification of farmland of slopes (Comprehensive Scientific Expedition, 1990), three typical slope gradients of 5 (S5), 15 (S15), and 25° (S25) were selected, representing slight, steep, and the steepest slopes, respectively. Three rainfall intensities of 60 (R60), 90 (R90), and 120 mm h^-1^ (R120) were selected, all within the range of typical erosive rainstorms in the Sichuan Basin. A total of 27 simulated rainfall events were conducted, incorporating three rainfall intensity levels and three slope gradients, with three replicates per treatment combination. The execution order of different treatment combinations (slope gradient × rainfall intensity) followed a completely randomized design.

Prior to performing the experiment, the soil flume was subjected to pre-rainfall with 30 mm h^-1^ intensity and 0° slope gradient until achieving continuous interflow. After pre-saturation, the soil flume was adjusted to the experimental slope. To prevent water evaporation, a plastic sheet was used to cover the soil flume and stand for 24 h.

During the rainfall simulation, the initial times of surface flow and interflow were first recorded. The surface flow and interflow samples were collected at the corresponding outlets every 2 min. The water samples were volumetrically measured, passed through a 0.65 μm filter membrane [34], and then filtered and analyzed immediately (or stored at 4°C in the dark for less than 24 hours before analysis) to prevent significant microbial alteration. All the simulated rainfall events were conducted in the laboratory under constant artificial light conditions to eliminate the influence of photodegradation.

### Laboratory analysis

The basic physicochemical properties of the tested soil, including soil bulk density; pH; soil organic carbon; total nitrogen, total phosphorus, total potassium; available nitrogen, available phosphorus, available potassium; and the distribution of particle size were all measured by China agrochemical analysis [35]. The dissolved organic carbon of water samples was determined using a TOC analyzer (Elementar Analysensysteme GmbH, Germany).

### Data analysis

The total DOC loss fluxes during a rainfall simulation event were calculated as follows:


$$Q=\frac{\sum_{i=1}^{n}\text{2}\times C_{i}\times R_{i}}{1000}$$ (1)

Where Q is the total DOC loss flux of surface flow or interflow in one rainfall simulation lasting for 1 hour, g m^-2^; C is the DOC concentration of surface flow or interflow, mg L^-1^; R is the runoff rate of surface flow or interflow, L m^-2^ min^-1^; n is the number of water samples of surface flow or interflow in one rainfall simulation. The coefficient 2 is the interval time of 2 minutes.

All statistical analyses were performed using the software package SPSS 20.0, and all the figures were prepared using Origin 9.0 software. Two-way analysis of variance (ANOVA) was used to detect the treatment effects on measured variables, and the Tukey’s Honest Significant Difference (Tukey HSD) was used to test comparisons among treatment means calculated at p < 0.05. Multiple linear regression was applied to investigate the relationships between DOC loss and rainfall intensity and slope gradient. Pearson’s correlations were used to study the correlations among the measured variables.

## Results

### Surface flow and interflow loss

The runoff initiation times of surface flow and interflow ranged from 0.85 to 4.47 min, and 2.16 to 3.70 min, respectively (Table 1). Both rainfall intensity and slope gradient exhibited accelerating effects on surface flow generation. For R60-S5 and R60-S15, the runoff initiation times of interflow were earlier than that of the surface flow, but under R90 and R120, the runoff initiation times of interflow lagged behind that of the surface flow. The basic physicochemical properties of the tested soil are displayed in Table 2.

**Table 1:** The characteristics of runoff under different rainfall intensities and slopes.

Rainfall intensity (mm h^-1^)	Slope gradient (°)	Initiation time of surface flow (min)	Initiation time of interflow (min)	Runoff rate of surface flow (L m^-2^ min^-1^)	Runoff rate of interflow (L m^-2^ min^-1^)
60	5	4.47	2.16	0.27 ± 0.05^c^	0.61 ± 0.06^a^
	15	3.21	2.87	0.45 ± 0.03^c^	0.34 ± 0.12^b^
	25	2.31	2.50	0.48 ± 0.06^c^	0.22 ± 0.05^b^
90	5	1.58	2.93	1.19 ± 0.15^b^	0.35 ± 0.08^b^
	15	1.15	3.70	1.50 ± 0.21^ab^	0.04 ± 0.03^c^
	25	0.85	3.50	1.48 ± 0.19^ab^	0.01 ± 0.02^c^
120	5	1.01	3.67	1.62 ± 0.15^ab^	0.02 ± 0.04^c^
	15	0.87	3.16	1.68 ± 0.15^ab^	0.02 ± 0.04^c^
	25	1.20	2.36	1.95 ± 0.16^a^	0.01 ± 0.05^c^

Note: different lowercase letters indicate significant differences at the *P* < 0.05 level.

**Table 2:** Basic physicochemical properties of the tested soil.

pH	BD (g cm^-3^)	SOC (g kg^-1^)	TN (g kg^-1^)	TP (g kg^-1^)	TK (g kg^-1^)	AN (mg kg^-1^)	AP (mg kg^-1^)	AK (mg kg^-1^)	Particle size (%)		
									Clay	Silt	Sand
8.42	1.31	10.09	1.06	0.79	20.22	35.13	28.38	120.00	12.5	62.3	25.2

BD: Soil bulk density, SOC: soil organic carbon, TN: total nitrogen, TP: total phosphorus, TK: total potassium, AN: available nitrogen, AP: available phosphorus, AK: available potassium.

**Fig 3:**
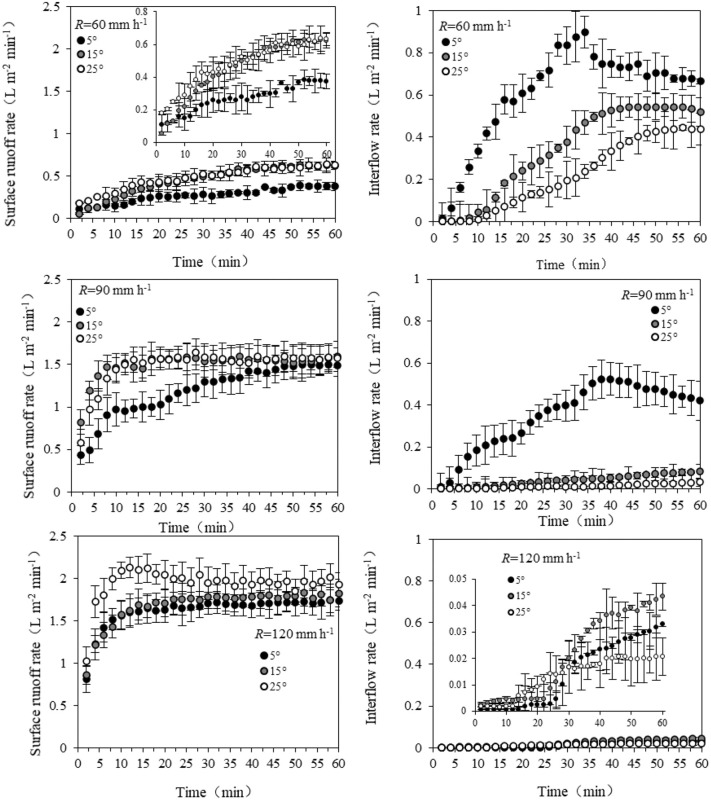
The effect of rainfall intensity and slope on the runoff processes. Hydrographs showing the surface runoff rate and interflow rate over time for rainfall intensities of 60, 90, and 120 mm/h and slope gradients of 5°, 15°, and 25°. Data are presented as mean ± SD (n=3).

The surface flow rate gradually increased with the rainfall duration, and then became stable with slight fluctuations (Fig 3). The time required to reach steady surface flow rate differed with different rainfall intensities and slopes. For low rainfall intensity or relative gentle slope (R60-S5, R60-S15, and R90-S5), the surface flow rates stabilized within 30−35 min, but for large rainfall intensity (R90 and R120) or steep slope gradient (S25), the surface flow rates sharply increased and stabilized within 10 minutes. The surface flow rates increased as the rainfall intensity increased. On average, the surface flow rates of R60 (0.27–0.48 L m^-2^ min^-1^) were significantly lower than those of R90 (1.19–1.50 L m^-2^ min^-1^) and R120 (1.62–1.95 L m^-2^ min^-1^) (*P* < 0.05), with no remarkable difference between R90 and R120 (*P* > 0.05, Table 1). Additionally, the slope gradient had no significant impact on surface flow rate, although the surface flow rate increased with the increase of slope.

The interflow curves were single-peak curves, showing trends of increasing initially and then decreasing under R60-S5 and R90-S5; the interflow rate gradually increased with rainfall duration under other treatments (Table 1). The interflow rates decreased with increased rainfall intensity, in the order of R60 (0.22–0.61 L m^-2^ min^-1^)> R90 (0.01–0.35 L m^-2^ min^-1^)> R120 (0.01–0.02 L m^-2^ min^-1^). The slope gradient had little effect on surface flow rates under high rainfall intensity.

### DOC concentration in surface flow and interflow

**Fig 4:**
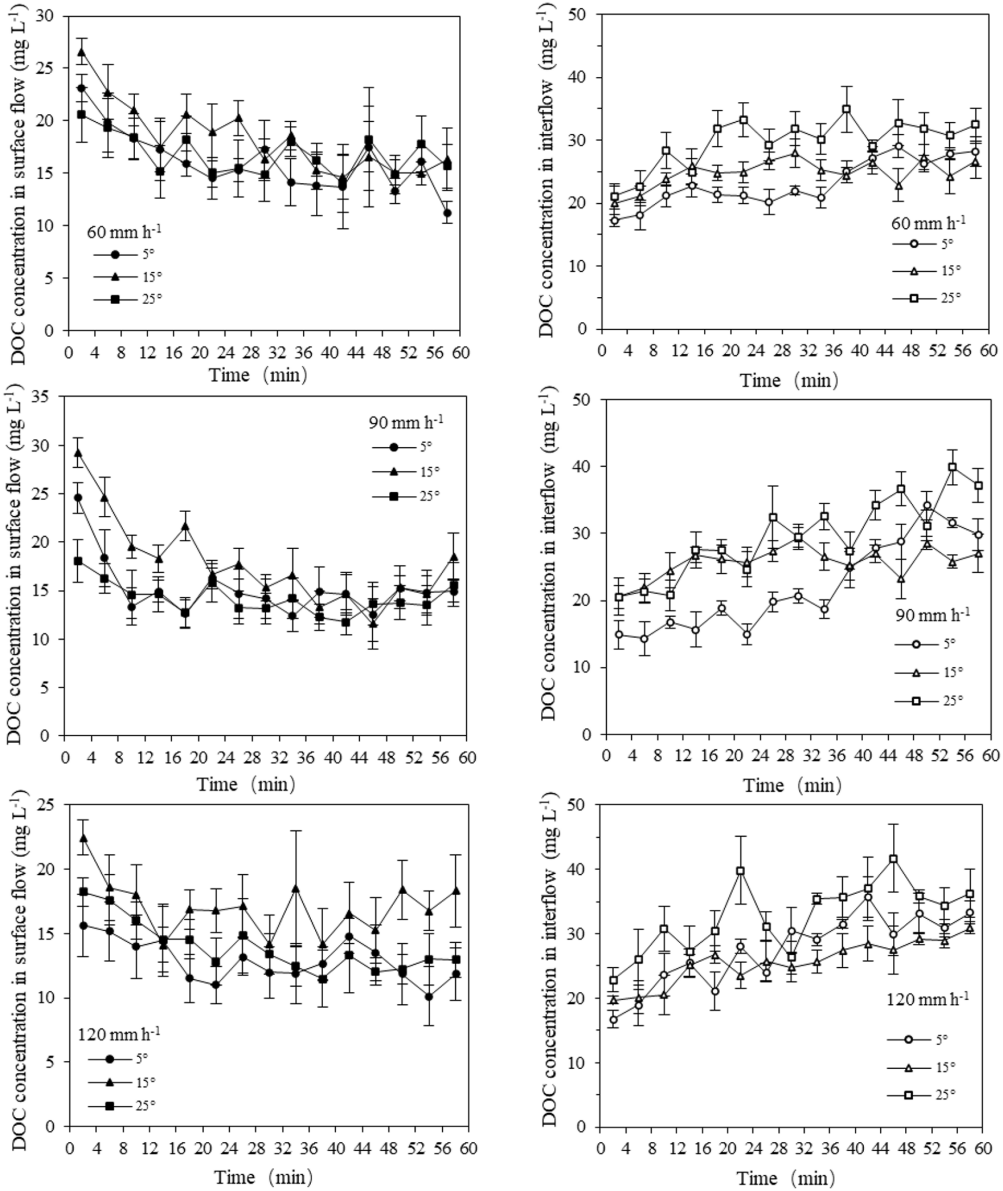
Changes in DOC concentration in the surface flow and interflow for different rainfall intensities and slopes. Temporal changes in DOC concentration for surface flow and interflow under rainfall intensities of 60, 90, and 120 mm/h on slopes of 5°, 15°, and 25°. Data are presented as mean ± SD (n=3).

As shown in Fig 4, the DOC concentration curves of surface flow peaked early and decreased gradually with the rainfall duration. The DOC concentration of surface flow decreased with the increase of rainfall intensities. The DOC concentrations of surface flow of R60, R90, and R120 ranged from 13.34 to 26.61 mg L^-1^, from 11.56 to 29.25 mg L^-1^ and from 10.11 to 22.44 mg L^-1^, respectively(Fig 4). The average DOC concentration of surface flow at the slope of 15° was always the highest, with 18.37 mg L^-1^ at 60 mm h^-1^, 17.84 mg L^-1^ at 90 mm h^-1^, and 17.00 mg L^-1^ at 120 mm h^-1^(Table 3).

**Table 3:** The DOC concentrations in the surface flow and interflow for different rainfall intensities and slope gradients.

Rainfall intensity (mm h^-1^)	DOC concentration in surface flow (mg L^-1^)			DOC concentration in interflow (mg L^-1^)		
	5°	15°	25°	5°	15°	25°
60	16.11 ± 2.09^Aa^	18.37 ± 2.09^Aa^	16.82 ± 2.4^Aa^	23.21 ± 1.57^Ab^	24.81 ± 1.97^Aab^	29.67 ± 2.54^Aa^
90	15.22 ± 1.89^Aa^	17.84 ± 1.85^Aa^	14.20 ± 1.85^Aa^	22.11 ± 1.8^Bb^	25.71 ± 1.95^Aab^	29.56 ± 2.41^Aa^
120	12.91 ± 2.05^Ab^	17.00 ± 2.29^Aa^	13.96 ± 1.8^Aab^	27.46 ± 2.23^Aa^	27.62 ± 2.03^Aa^	32.73 ± 3.28^Aa^

Note: the number after “±” is the standard deviation (SD); different capital letters in the same column indicate significant difference under different rainfall intensities (P < 0.05); different lowercase letters in the same row indicate significant difference under different slope gradients (P < 0.05).

The DOC concentration curves of interflow showed the opposite trends from the surface flow curves, and gradually increased with the rainfall duration (Fig 4). The concentration ranges of R60, R90, and R120 were 17.29–34.89 mg L^-1^, 14.32–39.93 mg L^-1^, and 16.75–41.71 mg L^-1^, respectively (Fig 4). For the low slope of S5, the average interflow DOC concentration at 120 mm h^-1^ was 27.46 mg L^-1^, significantly higher than that of R90 (Table 3). For the high rainfall intensity of R90 and R120, the slope gradient has a significant effect on the interflow DOC concentration. The DOC concentration of interflow increased when rainfall intensity increased, and the average DOC concentration of interflow at the slope of 25° was always the highest, with 29.56 mg L^-1^ at 90 mm h^-1^, and 32.73 mg L^-1^ at 120 mm h^-1^.

### Distribution of DOC loss fluxes via surface flow and interflow

**Fig 5:**
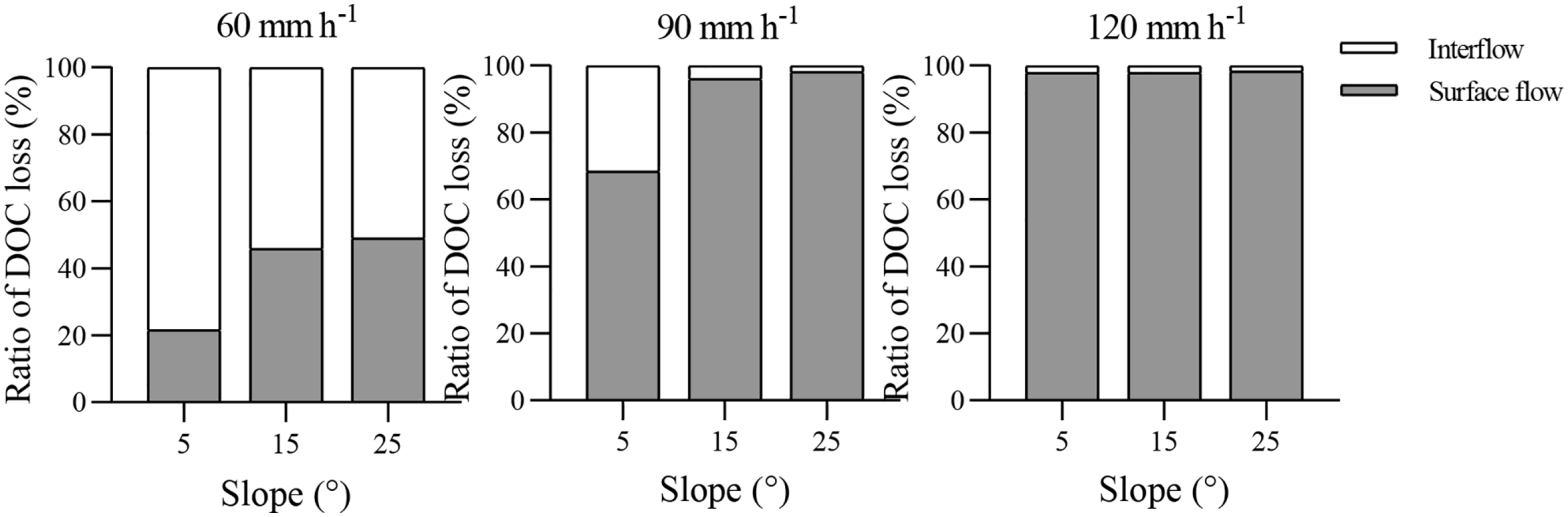
Distribution of DOC loss fluxes via surface flow and interflow. Figures display the ratios of surface flow and interflow DOC loss fluxes to the total DOC loss fluxes under rainfall intensities of 60, 90, and 120 mm/h on slopes of 5°, 15°, and 25°. The ratios of interflow DOC loss fluxes to the total DOC loss fluxes gradually decreased with the increase of rainfall intensity and slope gradient.

The DOC loss fluxes via surface flow for 60, 90, and 120 mm h^-1^ were 3.77–6.94 g m^-2^, 17.51–25.70 g m^-2^, and 19.69–26.94 g m^-2^, respectively (Table 4), and the corresponding DOC loss fluxes via interflow were 6.55–13.73 g m^-2^, 0.41–8.11 g m^-2^, and 0.42–0.55 g m^-2^, respectively. As the rainfall intensity and slope gradient increased, the DOC loss fluxes of surface flow increased. However, the opposite effect was seen for rainfall intensity and slope gradient on DOC loss fluxes of interflow. The highest DOC loss flux of the surface flow was 26.94 g m^-2^ for R120-S15 and smallest was 3.77 g m^-2^ for R60-S5. For the DOC loss fluxes of the interflow, the greatest was 13.73 g m^-2^ for R60-S5 and the smallest was 0.42 g m^-2^ for R120-S5. In addition, Fig 5 displays the ratios of surface flow and interflow DOC loss fluxes to the total DOC loss fluxes. Rainfall intensity and slope gradient had significant effects on the DOC loss distribution. The ratios of interflow DOC loss fluxes to the total DOC loss fluxes gradually decreased with the increase of rainfall intensity and slope gradient. For R60, interflow DOC loss percentages were 78.4%, 54.2%, and 51.0% at 5°, 15°, and 25°, respectively, all > 50%. For R120, the interflow DOC loss percentages were only 2.1%, 2.0%, and 1.7% at 5°, 15°, and 25°, respectively. According to the F-value and P-value (Table 5), slope gradient and rainfall intensity have a significant impact on surface flow DOC loss fluxes and interflow DOC loss fluxes (*P < 0.01*), and the influence of slope gradient is greater than that of rainfall intensity. Besides, the interaction between slope gradient and rainfall intensity has a significant impact on surface flow DOC loss fluxes (*P < 0.05*) and interflow DOC loss fluxes (*P < 0.01*), while the interaction between slope gradient and rainfall intensity has no significant impact on DOC concentration in surface flow and DOC concentration in interflow (*P > 0.05*).

**Table 4:** The DOC loss fluxes in the surface flow and interflow for different rainfall intensities and slope gradients.

Rainfall intensity (mm h^-1^)	Surface flow DOC loss fluxes (g m^-2^)			Interflow DOC loss fluxes (g m^-2^)		
	5°	15°	25°	5°	15°	25°
60	3.77 ± 0.18^Bb^	6.94 ± 0.31^Ba^	6.29 ± 0.53^Ba^	13.73 ± 0.71^Aa^	8.22 ± 0.35^Ab^	6.55 ± 0.27^Ab^
90	17.51 ± 0.76^Ab^	25.70 ± 1.42^Aa^	23.23 ± 0.97^Aa^	8.11 ± 0.52^Ba^	1.03 ± 0.08^Bb^	0.41 ± 0.06^Bb^
120	19.69 ± 1.34^Ab^	26.94 ± 1.01^Aa^	25.34 ± 0.37^Aa^	0.42 ± 0.02^Ca^	0.55 ± 0.03^Ba^	0.44 ± 0.01^Ba^

Note: the number after “±” is the standard deviation (SD); different capital letters in the same column indicate significant difference under different rainfall intensities (P < 0.05); different lowercase letters in the same row indicate significant difference under different slope gradients (P < 0.05).

**Table 5:** Two-way anova of DOC loss characteristics.

Factor	DOC concentration in surface flow		DOC concentration in interflow		Surface flow DOC loss fluxes		Interflow DOC loss fluxes	
	*F* value	*P* value	*F* value	*P* value	*F* value	*P* value	*F* value	*P* value
Rainfall intensity	2.18	*P >* 0.05	10.26	*P <* 0.01	797.00	*P <* 0.01	1181.00	*P <* 0.01
Slope gradient	3.91	*P <* 0.05	47.13	*P <* 0.01	81.42	*P <* 0.01	389.10	*P <* 0.01
Rainfall intensity*Slope gradient	0.26	*P >* 0.05	2.59	*P >* 0.05	4.911	*P <* 0.05	102.80	*P <* 0.01

### Influence factor correlation coefficient for DOC loss in surface flow and interflow

Multiple linear regression equations were developed among slope, rainfall intensity and DOC loss fluxes for surface flow and interflow (Table 6). Surface flow DOC loss fluxes were positively correlated with rainfall intensity and slope (R^2^ = 0.787, *P* < 0.01), however, interflow flow DOC loss fluxes were negatively correlated with rainfall intensity and slope (R^2^ = 0.822, *P* < 0.01). The partial correlation coefficients of rainfall intensity and slope were 0.881 and 0.426 for surface flow, and the corresponding absolute values of the partial correlation coefficients for interflow were 0.883 and 0.718. This indicated that the rainfall intensity has a greater impact on the DOC loss fluxes for both surface flow and interflow. Pearson analysis was performed and the correlations among rainfall intensity, slope gradient, runoff volume, and DOC loss via surface flow and interflow are displayed in Table 7. For surface flow, rainfall intensity and runoff volume were significantly correlated with DOC loss, with the highest correlation coefficient between runoff volume and DOC loss, 0.966, but insignificant correlations were seen between slope gradient and DOC loss. For interflow, rainfall intensity and slope gradient were negatively correlated with DOC loss, and runoff volume was positively correlated with DOC loss, with a correlation coefficient of 0.995. Additionally, the correlation between the three factors and DOC loss via surface flow and interflow were in the order of runoff volume>rainfall intensity>slope gradient.

**Table 6:** Regression equations for slope gradients (S), rainfall intensities, (I) and DOC loss in the surface flow and interflow.

Runoff type	Regression equation	R^2^	Partial correlation coefficient	
			Slope (°)	Intensity (mm h^-1^)
Surface flow	Y = 0.232S+0.305I-13.686	0.787	0.426	0.881**
Interflow	Y = −0.248S-0.15I + 21.643	0.822	−0.718*	−0.883**

**Indicates significant correlation at 0.01, *Indicates significant correlation at 0.05.

**Table 7:** Correlation analysis of slope, rainfall intensity and runoff with DOC loss in surface flow and interflow.

Factors	Surface flow DOC loss (g)	Interflow DOC loss (g)
Slope (°)	0.217	−0.436^*^
Rainfall intensity (mm h-1)	0.860^**^	−0.795^*^
Runoff volume (L)	0.966^**^	0.995^**^

** Indicates significant correlation at 0.01, * Indicates significant correlation at 0.05.

## Discussion

### Surface flow and interflow loss

Previous studies have shown that rainfall intensity and slope gradient are the most important factors affecting surface flow and interflow [22,36,37]. For the high rainfall intensity of R90 and R120 and steep slope gradient of S25, the surface flow rates sharply increased with the rainfall duration, and reached a steady state after a short-term increase. For R60-S5, R60-S15, and R90-S5, the surface flow rates slowly increased, which lasted a long-term, and reached a steady state until the end of rainfall (Fig 3). These results suggest that excess infiltration may be the main mechanism of surface flow under large rainfall intensity and steep slope gradient, but saturation may be the main cause of surface flow under low rainfall intensity and gentle slope gradient, results that are consistent with those of Li et al [11]. In this study, there are different trends of interflow rate for different rainfall intensities and slope gradients (Fig 3). Petry et al [38] reported that interflow includes preferential flow and matrix flow. Thus, further work should investigate the respective contributions of these two kinds of flow. In addition, defining the initial runoff time of the purple soil and its influencing factors should increase our understanding of the different mechanisms of runoff generation [12]. The initial time of surface flow decreased with the increase of rainfall intensity and slope (Table 1), but the change in the initial time of interflow was more complex. We concluded that rainfall intensity is the main factor affecting the initial time of interflow if the slope gradient is low. For a slope of 5°, the initial time of interflow is prolonged with increasing rainfall intensity. This finding is consistent with the results of Xin et al [39] who studied the purple soil area in southwest China.

**Fig 6:**
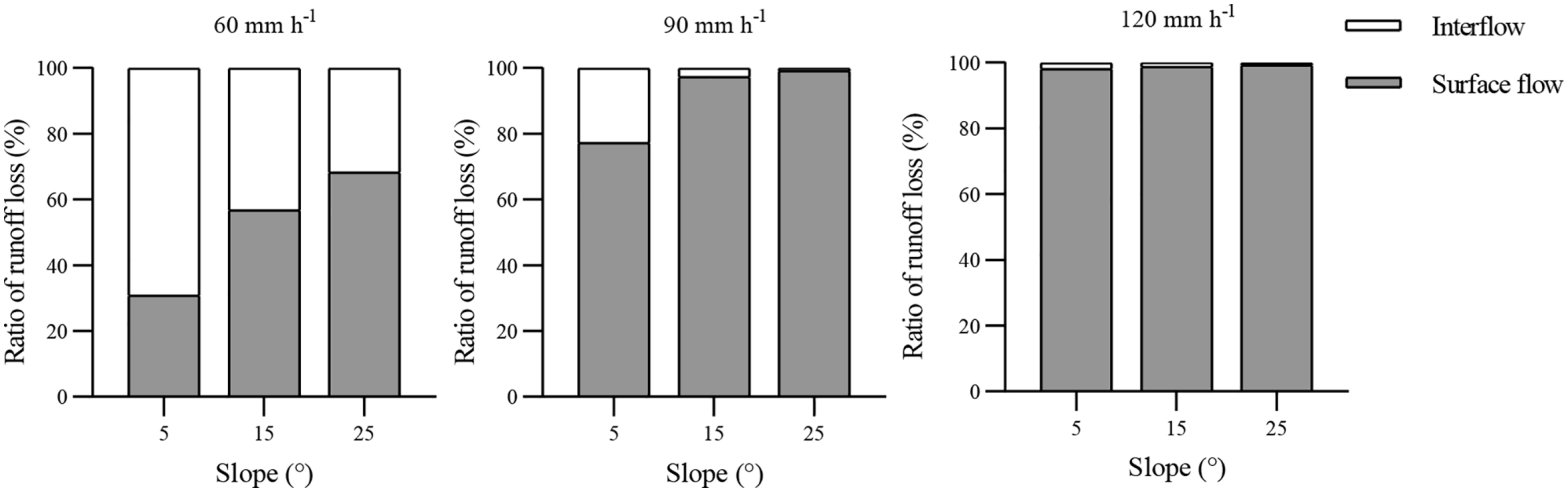
Distribution of surface flow and interflow volume. Figures display the ratios of surface flow and interflow loss to the total runoff loss under rainfall intensities of 60, 90, and 120 mm/h on slopes of 5°, 15°, and 25°. The ratios of interflow loss to the total runoff loss gradually decreased with the increase of rainfall intensity and slope gradient.

The observed distribution of surface flow and interflow indicates that rainfall intensity and slope gradient seriously affect the hydrological characteristics of purple soil slopes. As shown in Fig 6, the amount of interflow comprised a larger proportion of the total runoff volume under conditions of lower rainfall intensity and slope. Specifically, the interflow percentage under R60-S5 was the highest, 69.2%, and the interflow percentages decreased as the rainfall intensity and slope gradient increased, with 0.9–69.2%, 0.13–43.25%, and 0.7–31.9% for R60, R90, and R120, respectively. Hua et al [10] and Maïga-Yaleu et al [40] suggested that this phenomenon can be explained by water infiltration. The greater the rainfall intensity, the stronger the impact of raindrops. The splashing of raindrops can cause the sediment particles to clog the topsoil, forming a crust which was resistant to water infiltration. Therefore, the rate of interflow would be low under high intensity. Additionally, Wang et al [22] and Morbidelli et al [41] also reported that the infiltration rate decreased as the slope increased. However, the results differ from those of Fei et al [32], who reported that the interflow comprised the majority of the total runoff volume at steeper slopes, while the surface runoff comprised most of the runoff for gentle slope gradients of red soil. These differences may reflect differences in the soil types due to varied physical and chemical properties of the soil [33].

### DOC loss via surface flow and interflow

Nutrient loss through runoff is mainly affected by the amount of runoff volume and the solubility of nutrients in the runoff [15]. There are two pathways for DOC loss by runoff, namely, surface flow and interflow. Under identical rainfall intensities and slope gradients, the hydrological characteristics of surface flow and interflow are very different, with significant differences in DOC loss [25]. Fig 4 shows the trends of DOC concentration in the surface flow and interflow for different rainfall intensities and slope gradients. Opposite trends of DOC concentration were observed for the surface flow and interflow, consistent with the finding of Wang et al [22] that nitrogen concentration in the surface flow decreased with rainfall duration, but increased in the interflow. For surface flow, a large amount of soil organic carbon in the topsoil can be eroded away with increased rainfall duration, resulting in a gradual decrease of DOC concentration in surface flow, called a “flush effect” [42]. Besides, the 30 mm h^-1^ pre-event rainfall effectively “washes out” the readily leachable DOC that has accumulated at the soil surface and results in high initial concentrations of DOC and low concentration in surface flow [43]. For interflow, at the start of rainfall simulation, the interflow was dominated by preferential flow, with the macropores in the soil profile serving as channels of preferential flow lacking soil organic carbon [44]. Then the contribution of matrix flow gradually increased with rainfall duration, and adequate contact time between the infiltration water and the soil increased the dissolution of soil organic carbon in the interflow.

No significant differences were found in the surface flow-associated DOC concentration among the different rainfall simulations (*P* > 0.05), suggesting only small effects of rainfall intensity and slope gradient on the DOC concentration of surface flow (Table 3). The influence of the slope factor on the DOC concentration of the surface flow has been controversial [45,46]. In this study, the DOC concentration of surface flow was highest for a slope of 15°, 17.00–18.37 mg L^-1^. These results are similar to the findings by Wu et al [47] that the surface flow velocity increased with increased slope gradient, and the finding of an increase in the dissolution of soil nutrients. However, at a critical slope gradient, the erosion transport capacity would decrease with increased slope gradient. Fei et al [32] explained that this switch could be attributed to breakdown of soil aggregates. For a gentle slope, raindrops can damage small soil aggregates, releasing soluble organic matter to increase DOC concentration of runoff. With a steeper slope gradient, large aggregates with lower soil organic carbon content tend to move more, thereby decreasing the DOC concentration of the surface flow [48]. No significant differences in interflow DOC concentrations were also observed for the different rainfall intensities. Overall, for both surface flow and interflow, the rainfall intensity had little effect on DOC concentration, consistent with the findings of Jin et al [21]. We also found that the effect of slope gradient on DOC concentration of interflow was only significant with a rainfall intensity of 120 mm^-1^ with the lowest interflow DOC concentration for the 15° slope. This is because a large proportion of the topsoil enriched in organic carbon is eroded away by surface flow, with dissolving and infiltration of only a small fraction of the soluble organic carbon into the soil, resulting in a low concentration of interflow.

As shown in Table 4, the DOC loss fluxes of surface flow ranged from 3.77–26.94 g, and those of interflow varied from 0.41–13.73 g. For R60, the DOC loss fluxes of interflow were 78.4%, 54.2%, and 51.0% of total DOC loss at 5°, 15° and 25°, respectively.These results indicated that the interflow is a crucial route of DOC loss under low rainfall intensity and low slope gradient, so nutrient loss through this pathway is significant for in purple soil area, a finding that is consistent with that of Hua et al [29]. In contrast, Kindler et al [6] argued that the DOC loss caused by interflow was comparatively small. This is because that DOC transport associated with interflow is a complex hydrological process that is determined by the amount of DOC in soil, the adsorption and desorption processes of DOC, and the physical and chemical properties of the soil [49].

### Influence factor correlation coefficient for DOC loss in surface flow and interflow

**Fig 7:**
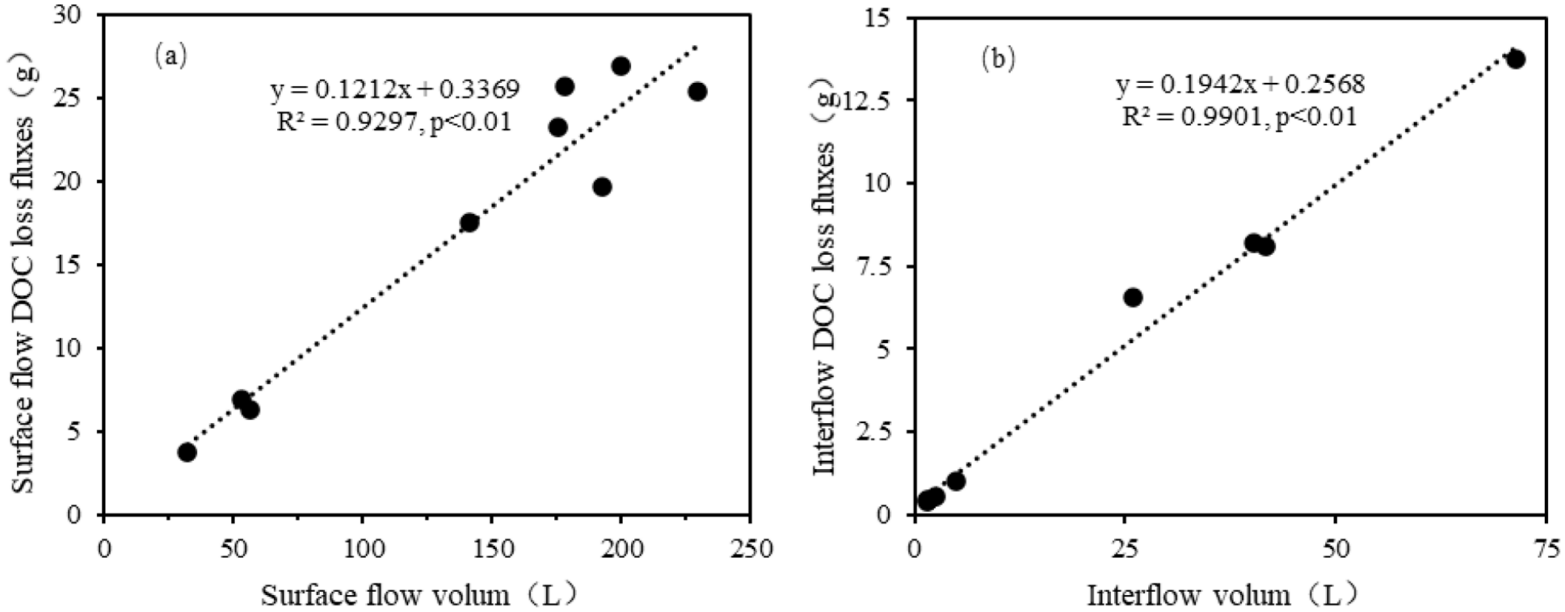
Runoff-associated DOC loss as a function of surface flow (a) and interflow volum (b) under different rainfall intensities and slope gradients. Scatter plots show the linear relationship between DOC loss fluxes and (a) surface runoff volume (R^2^ = 0.93) and (b) interflow volume (R^2^ = 0.99). The dotted lines represent the linear regression fits for each flow pathway.

The DOC loss fluxes can be influenced by many factors, including rainfall intensity, slope gradients, surface runoff, and interflow [50]. According to Pearson analysis (Table 7), the influence factor correlation coefficients for DOC loss in surface flow and interflow were ranked as follows: runoff volume>rainfall intensity>slope. Additionally, runoff volume exhibited a positive linear correlation with corresponding DOC loss fluxes in the surface flow (R^2^ = 0.93, *P* < 0.01) and the interflow (R^2^ = 0.99, *P* < 0.01), respectively (Fig 7). Thus, to control DOC loss via runoff, runoff volume should first be considered, followed by rainfall intensity and slope gradient. Previous studies showed that soil and water conservation practices, including contour ridges, straw mulching, and canopy interception of precipitation, can effectively reduce the amount of surface flow by promoting water infiltration [51,52]. However, at the same time, the volume of interflow probably increased in well-developed interflow soils [53]. Here, the DOC concentrations in the interflow were 1.35–2.34 times higher than those of the surface flow (Table 3), suggesting that reducing the amount of surface flow may increase the risk of DOC loss through interflow under unreasonable measures. Therefore, additional research is needed to investigate the effects of prevention and control practices to reduce nutrient loss in purple soil area.

## Conclusions

Rainfall simulation experiments of purple soil were conducted under three rainfall intensities (60, 90, and 120 mm h^-1^) and three slope gradients (5, 15 and 25°) to investigate runoff and DOC loss processes. The results showed that increasing the rainfall intensity and slope significantly accelerated surface flow generation and increased the surface flow rates, but delayed interflow generation and decreased interflow rates. The DOC concentration in the surface flow decreased with rainfall duration, and the opposite trend was seen for DOC concentration in interflow. For both surface flow and interflow, the rainfall intensity and slope gradient had little effect on DOC concentration, while these two factors affect DOC loss fluxes mainly by affecting the runoff rate of surface flow and interflow. Thus, the effects of rainfall intensity and slope gradient on DOC loss fluxes via surface flow and interflow were similar to the effects on the runoff rate of surface flow and interflow, that is, DOC loss fluxes of surface flow increased with increased rainfall intensity and slope gradient, and DOC loss fluxes of interflow decreased. In addition, interflow is a major hydrological pathway of DOC loss under low rainfall intensity and gentle slope gradient, but surface flow is more important under high rainfall intensity and steep slope gradient. Overall, the results indicate the lateral transport of DOC via interflow should be further studied to control carbon loss and enhance carbon sequestration in purple soil area.

## Supporting information

S1 File Data. (XLSX)
